# Concerns about disclosing a high-risk cervical human papillomavirus (HPV) infection to a sexual partner: a systematic review and thematic synthesis

**DOI:** 10.1136/bmjsrh-2019-200503

**Published:** 2020-01-08

**Authors:** Kirsty F Bennett, Jo Waller, Mairead Ryan, Julia V Bailey, Laura A V Marlow

**Affiliations:** 1 Cancer Communication and Screening Group, Department of Behavioural Science and Health, University College London, London, UK; 2 Cancer Prevention Group, School of Cancer and Pharmaceutical Sciences, King's College London, London, UK; 3 e-Health Unit, Department of Primary Care and Population Health, University College London, London, UK

**Keywords:** cervical screening, human papillomavirus, sexually transmitted infections

## Abstract

**Background:**

Human papillomavirus (HPV)-based cervical screening is now replacing cytology-based screening in several countries and many women in screening programmes will consequently receive HPV-positive results. Because of the sexually transmitted nature of HPV, receiving an HPV-positive result may raise questions about disclosing the infection to a sexual partner.

**Objective:**

To review the quantitative and qualitative literature exploring women’s concerns about disclosing a high-risk cervical HPV infection to a sexual partner.

**Methods:**

We searched MEDLINE, PsycINFO, CINAHL Plus, Web of Science and EMBASE for studies reporting at least one disclosure-related outcome among women with high-risk HPV. We also searched the grey literature and carried out forward/backward citation searches. A narrative synthesis for quantitative studies and a thematic synthesis for qualitative studies were conducted.

**Results:**

Thirteen articles met the inclusion criteria (12 qualitative, 1 quantitative). In the quantitative study, 60% of HPV-positive women felt disclosing an HPV result was ‘risky’. Concerns about disclosing HPV to a sexual partner were influenced by the stigma that is associated with having an STI and uncertainty about how their partner would respond. Women questioned how, when and to whom they should disclose their HPV-positive status.

**Conclusions:**

The studies included in this review provide rich information about the range of concerns women have, the reasons for these concerns, and the questions women have about disclosing HPV to sexual partners. As studies were predominantly qualitative, the prevalence of concerns is unclear.

**Key messages**

- This is the first review to synthesise the literature on women’s concerns about disclosing a high-risk cervical HP Vinfection to a sexual partner.
- This review identified that concerns about disclosing HPV to a sexual partner are partly because of the stigma associated with having an STI and uncertainty about how a partner might respond.
- Some women have questions about disclosure including who they should disclose to and how to approach and manage these conversations.
- Increasing knowledge of the high prevalence of HPV and providing clear information in screening letters and leaflets about disclosing may help women understand their screening result and minimise unnecessary concerns.

## Introduction

Virtually all cervical cancers are caused by persistent infection with a high-risk type of human papillomavirus (hrHPV).^1–3^ HPV is a sexually transmitted infection (STI)^4^ which affects both men and women, and it has been estimated that 80% of individuals will acquire a genital HPV infection by the age of 50 years.^5^ There are many types of HPV and these are divided into low-risk types (which do not cause cancer but can cause genital warts or verrucas) and high-risk types (which can cause cells to become abnormal and, over time, can lead to cancer if left untreated). While infection with hrHPV is the underlying cause of almost all cervical cancers, hrHPV rarely causes cancer and most infections resolve spontaneously within 2 years.^6^

Until recently, most cervical screening programmes in high-income countries used cytology to detect cervical abnormalities, with HPV testing used as a triage for women with borderline or low-grade cell changes.^7^ However, using HPV testing as the primary test in cervical screening has higher sensitivity for detecting high-grade cervical abnormalities^8–10^ and as a result several countries have moved, or plan to move, to primary HPV testing.^11–13^ In England, primary HPV testing in the NHS Cervical Screening Programme will be fully rolled-out by the end of 2019. In a screening programme that uses primary HPV testing, women who test positive for hrHPV will be told they have HPV alongside receiving a normal or abnormal cytology result.^14^

Research suggests that a key concern among individuals with an STI is disclosing their diagnosis to a sexual partner. In studies with participants with herpes simplex virus (HSV) and chlamydia, disclosure is described as something that is difficult, fear-inducing^15^ and a considerable source of worry.^16^ This may be due to the feelings of stigma and shame that are associated with having an STI,^17–19^ which has been found to be a barrier to disclosing some STI diagnoses.^19^ Participants’ concerns about disclosure include worry that they will receive a negative reaction from their partner,^16 20–22^ concern about being rejected by their partner,^20 23^ or that their partner will end their relationship^20^ and worry that their partner would inform others of the infection.^20 22^ An early qualitative study of HPV testing in cervical screening suggested that some women with HPV have concerns about disclosing an HPV-positive test result to their partner.^24^

Contact tracing (identifying individuals who have come into contact with an infected individual) is important for some STIs so that previous partners can be screened and treated for the infection if necessary. However, there is no treatment for HPV and the World Health Organization (WHO) advise against routine contact tracing for HPV.^25^ Therefore, the decision about whether to disclose HPV to a sexual partner is a personal choice. It is important to understand women’s information needs around disclosure so that these can be met through information provision and guidance from healthcare professionals. We reviewed the quantitative and qualitative literature exploring women’s concerns about disclosing a high-risk cervical HPV infection to a sexual partner.

## Methods

This review was registered with PROSPERO (CRD42018083969) and followed the Preferred Reporting Items for Systematic Review and Meta-Analysis (PRISMA) guidelines.^26^ The review explored two research questions with findings reported separately. Details of the methods used for both reviews are reported in full elsewhere.^27^

### Search strategy for identifying papers

We searched MEDLINE, PsycINFO, CINAHL Plus, Web of Science and EMBASE on 9 January 2019. The search included terms relating to (i) high-risk cervical HPV and (ii) a psychosexual or disclosure-related outcome (eg, sexual behaviour, sexual function, disclosure of HPV status to a partner) and were linked using Boolean operators (see online supplementary material 1 for the full search strategy). Both qualitative and quantitative papers were eligible for inclusion and no study design, date, or language limits were applied to the initial search. We also searched the reference lists of included articles, conducted forward citation searching and searched the grey literature using OpenGrey (www.opengrey.eu) to identify any additional eligible articles.

10.1136/bmjsrh-2019-200503.supp1 Supplementary data

### Study selection process

The titles of all articles identified from the search were screened by one reviewer (KFB). Two reviewers (KFB and MR) screened the abstracts of the remaining articles. Articles were included if they mentioned (i) HPV and (ii) a psychosexual or disclosure-related outcome. Reviews, conference abstracts, commentaries, opinion pieces and editorials were excluded. Articles were also excluded if they were not written in English, focused on the psychosexual impact of cervical cancer, or treatment for cervical cancer or colposcopy. We decided not to include articles that focused exclusively on low-risk types of HPV (ie, genital warts) because (i) primary HPV testing will be for high-risk types of HPV and (ii) feelings about disclosing low-risk HPV were expected to be distinct because of its symptomatic, visible nature. Full-texts were obtained where an article could not be assessed from the abstract. Disagreements were resolved by discussion.

### Data extraction

Data were extracted from each article using a standardised data extraction form (see online supplementary material 2). Extracted data included participant characteristics, study methods and a summary of disclosure-related outcomes. One reviewer (KFB) extracted information from each article with a second reviewer (MR) independently extracting information for 30% of the studies. Inconsistencies were resolved through discussion.

10.1136/bmjsrh-2019-200503.supp2 Supplementary data

### Quality assessment

A quality assessment was carried out for each article using modified versions of the National Institute for Health and Care Excellence (NICE) quality appraisal checklists for quantitative and qualitative studies (see online supplementary material 3 and 4). One reviewer (KB) carried out the quality assessments with a second reviewer (MR) independently conducting 30% of assessments. The agreement rate between reviewers was 75%. Disagreements about study quality were resolved through discussion.

10.1136/bmjsrh-2019-200503.supp3 Supplementary data

10.1136/bmjsrh-2019-200503.supp4 Supplementary data

### Analysis

For qualitative studies we conducted a thematic synthesis, following three stages outlined by Thomas and Harden:^28^ (1) line-by-line coding of text in the results and discussion sections according to the meaning and content, (2) identifying ‘descriptive themes’ by looking for similarities and differences between codes and beginning to group them together into a hierarchy, (3) and generating ‘analytic themes’ which involves going beyond the content of the studies to generate new interpretive constructs or explanations. One author (KFB) developed a coding frame and applied it to the data with a second reviewer (MR) independently coding 25% of the included articles. Any inconsistencies were resolved through discussion. There was only one quantitative study which has been reported descriptively.

## Results

### Search results

**Figure 1:**
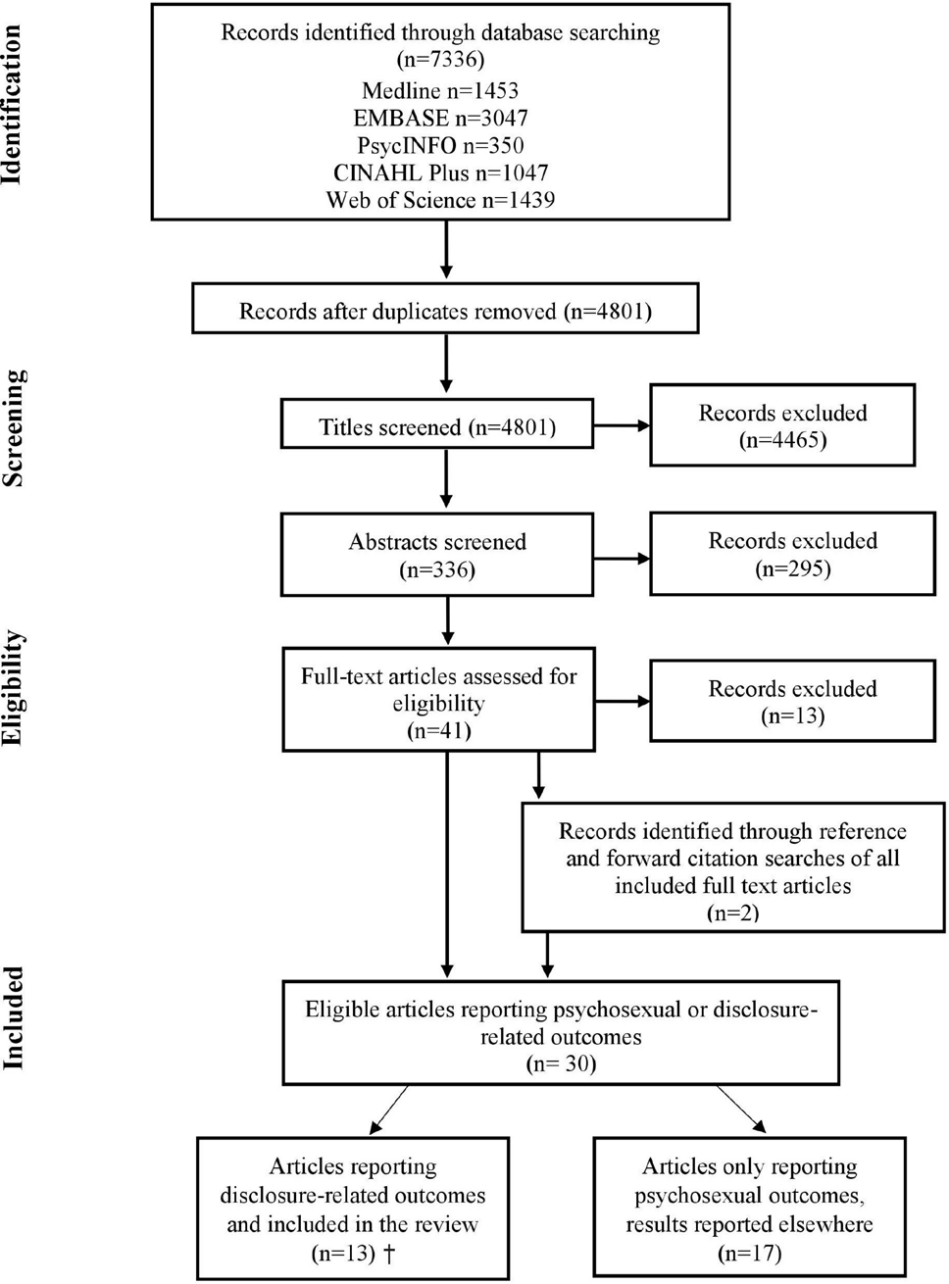
Flow diagram of study selection (adapted from Moher *et al* 26) Of the 13 articles included in this review, eight articles included both disclosure and psychosexual-related outcomes and are reported in this article and elsewhere.^27^

The initial search returned 7336 articles, which reduced to 4801 after the removal of duplicates. Of these, 4465 were excluded on the basis of their title, leaving 336 abstracts to be reviewed. Following exclusions, 41 full-texts were reviewed. Thirteen articles were excluded during the full-text review and an additional two articles were identified following backward and forward citation searches, resulting in 30 papers (see figure 1). Thirteen studies assessed concerns about disclosing an HPV infection to a sexual partner and are included in this analysis.^24 29–40^ figure 1 shows the study selection process.

Studies were conducted in the US (n=7), UK (n=2), Australia (n=2), Taiwan (n=1) and Brazil (n=1) and were published between 2005 and 2016. Studies were predominantly qualitative (n=12),^24 29 30 32–40^ with one quantitative study.^31^ Most studies collected data using individual interviews (n=11).^24 30 32–40^ One qualitative study^29^ collected patient narratives of having HPV from a website of patient experiences and analysed these using content analysis. Participant and study characteristics are shown in table 1.

**Table 1:** Characteristics of studies measuring disclosure-related outcomes included in the review

Reference	Country	Years study conducted	Age (years)	Participants (n)	Study design	Study population	Disclosure outcomes reported
Daley *et al* (2015)^31^	USA	2003–2005	Men: 18–66 Women: 18–65	344	Questionnaire completed following receipt of an HPV-positive result	Women (n=154) attending a student health service clinic and planned parenthood clinics for a gynaecological examination and Pap smear Men (n=190) participating in the HPV in men study (HIM)*	Anticipated psychological impact of disclosure
Barnack-Tavlaris *et al* (2016)^29^	USA	2013	Not specified	127 blog posts	Content analysis of HPV blog posts	Individuals who posted a blog to the *Experience Project* website experience of “I have HPV”	Anticipated psychological impact of disclosure
Bertram & Magnussen (2008)^30^	USA	Not specified	18–65	10	Unstructured interviews	Women with a history of an abnormal Pap smear recruited at the time of their annual gynaecological examination from a women’s health clinic in Hawaii	Anticipated psychological impact of disclosure, when is disclosure necessary?, managing disclosure
Kosenko *et al* (2012)^32^	USA	Not specified	19–56	25	Semi-structured interviews	Women answering an advertisement posted online (on social media websites and online support groups) and in community centres, libraries, restaurants, coffee shops, supermarkets and buildings in college campuses in cities in the southeastern United States about the stress and coping of women with HPV	Anticipated psychological impact of disclosure, managing disclosure
Kahn *et al*. (2005)^33^	USA	2002	14–21, mean: 17.2	100	Individual interviews	Women attending an urban, hospital-based teen health centre who were tested for HPV	Anticipated psychological impact of disclosure
Lin *et al* (2011)^34^	Taiwan	2008	27–56	20	Semi-structured interviews	Women attending a gynaecological outpatient clinic of a university-based hospital in Taipei, Taiwan	When is disclosure necessary?, managing disclosure
McCaffery & Irwig (2005)^35^	Australia	2002	Range unknown, 53% were <35 years, 47% were >35 years	19	In-depth, unstructured interviews	Women attending family planning clinics, general practice and specialist gynaecologist practices in Sydney, Australia, and the surrounding area	Anticipated psychological impact of disclosure, When is disclosure necessary?
McCaffery *et al* (2006)^24^	UK	2001–2003	Age categories reported: 20–29, 30–39, 40–49, 50–64	74	In-depth interviews	Women taking part in clinic clinical trials of HPV testing or attending colposcopy clinics where HPV testing is carried out	Anticipated psychological impact of disclosure, when is disclosure necessary?, managing disclosure
McCurdy *et al* (2011)^37^	USA	2003–2004	18–47 (women that the article focuses on were aged between 21 and 45)	42 (article focuses on 18 women who were aware of their HPV status)	In-depth interviews	Women attending three private primary care clinics who were found to have atypical squamous cells of undetermined significance (ASCUS) or a low-grade squamous intraepithelial lesion as well as a high-risk HPV type	Anticipated psychological impact of disclosure
Newton & McCabe (2008)^38^	Australia	Not specified	19–59	60 (30 with genital herpes, 30 with HPV)	Semi-structured interviews	Men (n=30) and women (n=30) responding to an advertisement about the study posted on STI websites, support groups and online STI communities	Anticipated psychological impact of anticipated disclosure, when is disclosure necessary?
Parente Sa Barreto *et al* (2016)^39^	Brazil	2012	20–42	14	Semi-structured interviews	Women attending a Specialised Medical Carer Service unit (a public service supporting sexual and reproductive care). Women were excluded from the study if they were attending the unit for the first time	Anticipated psychological impact of disclosure, managing disclosure
Perrin *et al* (2006)^40^	USA	Not specified	18–44	52	In-depth, semi-structured interviews	Women diagnosed as having one or more types of HPV attending one of three clinical sites (two Planned Parenthood clinics or the Student Health Service clinic at the University of South Florida) for an annual gynaecological examination	Anticipated psychological impact of disclosure, managing disclosure
Waller *et al* (2007)^36^	UK	2003	21–64	30	In-depth, semi-structured interviews	Women taking part in the ARTISTIC trial of HPV testing (a randomised trial of HPV testing in primary cervical screening)	Anticipated psychological impact of disclosure

*The focus of this review was women’s concerns about disclosing HPV and therefore the findings from men taking part in this study were not included in the review.

HPV, human papillomavirus; STI, sexually transmitted infection.

### Quality assessment

All qualitative studies were judged to be well conducted. The single quantitative study was judged to have been designed or conducted in such a way as to minimise the risk of bias and had good internal and external validity (see table 2 for details).

**Table 2:** Quality assessment rating for studies included in the review

Study	Internal validity*	External validity*	Overall assessment score†
Daley *et al* (2015)^31^	‡	§	
Barnack-Tavlaris *et al* (2016)^29^			‡
Bertram & Magnussen (2008)^30^			‡
Kosenko *et al* (2012)^32^			‡
Kahn *et al*(2005)^33^			‡
Lin *et al* (2011)^34^			§
McCaffery & Irwig (2005)^35^			‡
McCaffery *et al* (2006)^24^			‡
McCurdy *et al* (2011)^37^			‡
Newton & McCabe (2008)^38^			§
Parente Sa Barreto *et al* (2016)^39^			§
Perrin *et al* (2006)^40^			‡
Waller *et al* (2007)^36^			‡

*For quantitative studies.

†For qualitative studies.

‡Indicates that the study was designed or conducted in such a way as to minimise the risk of bias.

§Indicates that the study was partly designed to minimise bias, may not have addressed all potential sources of bias, or it was not clear from the way the study was reported.

¶Indicates that the study had significant sources of bias across all aspects of the study design.

### Qualitative studies

We conducted a thematic synthesis of the 12 qualitative studies that assessed concerns about disclosing an HPV infection to a sexual partner. Three major themes were identified: (i) Anticipated psychological impact of disclosure, (ii) When is disclosure necessary? and (iii) Managing disclosure. Table 3 gives a brief description of each theme and provides additional example quotes.

**Table 3:** Brief description of themes relating to the psychological impact of disclosing a human papillomavirus infection to a sexual partner and the studies associated with them

Theme	Subtheme	Studies	Explanation	Quote(s)
Anticipated psychological impact of disclosure	General concerns about disclosure	Barnack-Tavlaris *et al* 29 Bertram & Magnussen^30^ Kosenko *et al* 32 McCaffery *et al* 24 McCurdy *et al* 37 Newton & McCabe^38^	Women reported feeling anxious, worried and fearful about disclosing HPV to a sexual partner.	*"For some, the stress of disclosure appeared to be the most difficult aspect of managing the HPV infection."* [A]^24^ *"I feel apprehensive about having to disclose this information to a sexual partner: I know that I will feel vulnerable at that moment."* [P]^38^
	Stigma of having an STI	Bertram & Magnussen^30^ McCaffery *et al* 24 McCurdy *et al* 37 Perrin *et al* 40 Waller *et al* 36	Women were concerned about disclosing the infection because of the perception of promiscuity that is associated with having an STI.	*"Feelings of shame and stigma associated with having an STI may affect willingness to disclose HPV to a sexual partner."* [A]^40^ *"The stigma of HPV as a sexually transmitted infection was more devastating to some than the fear of cancer."* [A]^30^
	How will others respond?	Barnack-Tavlaris *et al* 29 Kosenko *et al* 32 Kahn *et al* 33 McCaffery & Irwig^35^ McCaffery *et al* 24 McCurdy *et al* 37 Newton & McCabe^38^ Parente Sa Barreto *et al* 39	Women were concerned how their partner would respond to disclosure, for example, whether their partner’s perception of them would change or that a partner might reject them (sexually or by ending the relationship).	*"What about when I tell a guy I want to be with that I have HPV? Will he run away as if I'm some dirty girl that sleeps around, which I'm anything but?"* [P]^29^
When is disclosure necessary?		Bertram & Magnussen^30^ Kosenko *et al* 32 Lin *et al* 34 McCaffery & Irwig^35^ McCaffery *et al* 24 McCurdy *et al* 37	Women questioned whether it was necessary to disclose, particularly to male partners, as women were unsure of the impact for them. Women also questioned to whom they should disclosure to and the best time to disclose.	*"I guess there aren’t many repercussions for the male partner. That is the hardest part: it’s the partner piece. That was the biggest issue. It was really hard to find any information on it* [HPV in men] *even to find something that says it won’t affect them."* [P]^30^ *"It’s not like I had tons of partners, but it really could’ve been any of them. I don’t know when, I don’t know where, I don’t know who. I don’t know who I’m supposed to tell…".* [P]^32^
Managing disclosure		Bertram & Magnussen^30^ Kahn *et al* 33 Lin *et al* 34 McCaffery *et al* 24 Perrin *et al* 40	Some women chose to focus on the abnormal cervical screening result rather than on testing positive for HPV.	*"I have told my partner that they don’t know where it comes from … obviously because he’d look at me in a different light because … he’d be like, have I got it or has she been with someone else?"* [P]^24^ *"To manage the anxiety many women chose not to tell their partner about their HPV infection, instead focusing on their abnormal cytology result which did not carry direct connotations of sexual transmission."* [A]^24^

[P] denotes a participant comment; [A] denotes an author comment. Superscript number in the Quote(s) column denotes the number of the study in the reference list.

HPV, human papillomavirus; STI, sexually transmitted infection.

### Anticipated psychological impact of disclosure

The first theme describes the thoughts, feelings and concerns women had prior to disclosing HPV to a sexual partner. In addition to expressing general concerns, women were concerned about the stigma that was attached to having an STI and how their partner would respond.

#### General concerns about disclosure

While some women were not worried about disclosing the infection, others felt that the prospect of disclosure was challenging, complicated and something they wished to avoid. Women were often anxious, worried, fearful and stressed about discussing HPV with their sexual partners:^24 29 30 32 37 38^

*"The thought of having it, deciding when to do it and how and what to say - it was extremely stressful"* [P = participant comment].^32^

Their concerns about disclosing were partly due to the stigma and shame associated with having an STI and how others would respond. Concern that they may have transmitted the infection to their partner and perceptions that partners had a poor understanding of HPV also enhanced anxiety around disclosure:^24 37^ *"Women repeatedly described feeling highly anxious about informing their partner, with descriptions of 'bursting into tears' and feeling intensely 'guilty' and worried that they may have infected their partner with the virus"* [A = author comment].^24^ Feeling depressed about having to disclose the infection to sexual partners was reported, although this was uncommon.^38^

#### The stigma of having an STI

Women’s concerns about anticipated disclosure were partly due to the stigma of having an STI. Women were apprehensive that they might be viewed as being promiscuous.^29 32 37^ For some, the stigma of having an STI had a greater impact than concern about cancer.^30^ Women felt ashamed and embarrassed about having an STI,^24 37^ and the authors of one paper reported that these feelings may affect willingness to disclose an HPV infection to a sexual partner.^40^

#### How will others respond?

Concerns about how others would respond and react to HPV disclosure seemed to be influenced by the negative connotations of having an STI. Women were concerned that their partner would perceive them differently:^24 35^ "*I was more worried about my partner reading it and saying 'aha'. I was worried about him thinking it was sexually transmitted and that I picked it up before I met him which would have concerned him a lot as we had only been together about 4 or 5 months at that stage… I was worried that it might change his opinion of me and being early in a relationship* [it was a] *bit of a concern"* [P].^35^ There were concerns about being rejected by a partner following disclosure^29 33 38^ and some women had specific concerns that they might be sexually rejected: *"If I told men that I had it they might not want to have sex with me"* [P].^24^ Some women were worried that their partner would accuse them of infidelity^37 39^ and felt that disclosure might cause harm to their relationship or even lead to it ending.^33 37 39^ In extreme cases, women ended relationships because of a fear of rejection following disclosure.^38^

### When is disclosure necessary?

The second theme related to women’s views on whether it was necessary to disclose their HPV-positive status to a sexual partner. While some women felt obligated to disclose the infection to current and prospective partners,^30 32^ a common response was to question whether it was necessary to disclose to current and previous partners.^24 30 34 35 37^ This was often due to the perceived lack of serious physical consequences of HPV for men.^24 30 34^ A lack of clear, consistent information also led women to question whether it was necessary to disclose:^24^ "*Should I be telling sexual partners that I have this? And one person would say yes of course you must and another would say don’t be silly almost all the population’s been exposed to it … I couldn’t get to the truth … they were giving me conflicting advice … I found that very distressing that I couldn’t actually get real information that I could trust"* [P]. 24

### Managing disclosure

The third theme related to managing disclosure. Some women reported that they were uncertain about how to approach disclosure^32 34^ and wondered about the most appropriate time to disclose:^32^ "*It's always in the back of your head. You know, 'Is he going to ask me back to his place? If he does, should I tell him?' It was just, 'When do I tell him?'… So, it was very much like 'What's the best timing?'… It was a lot of planning and stressing out and asking my friends, 'Do you think I need to tell him?'"* [P].^32^ Some women chose not to disclose their HPV result and instead chose to tell their partners about their abnormal cytology result, potential cervical cancer, or having a gynaecological disease.^24 30 34^ This was seen as a way to minimise anxiety^24^ and avoid the embarrassment or complication of explaining about HPV.^24 30^ Other women described being deliberately vague about how HPV was transmitted because they were concerned about how their partner would react.^24^ Some chose not to disclose the infection to male partners because they perceived that HPV did not have an impact or did not know what to tell their partner.^24^ The authors of one paper describe the decision not to disclose as being "… *motivated by women’s desire to minimise their own anxiety during an already stressful period and to avoid dealing with a difficult issue of which they had only limited understanding" [*A*]*.^24^

#### Quantitative study

Only one quantitative study reported outcomes relating to disclosure of HPV to a sexual partner.^31^ HPV-positive women (n=154) aged 18–45 years were recruited through student health services and planned parenthood clinics in Florida and were asked to complete a paper survey about negative emotions (eg, anger, worry, confusion) and HPV-related stigma beliefs in relation to their HPV test result. A single statement assessed feelings about disclosure: ‘Disclosing my HPV test result is risky’, with 60% of women agreeing with this statement.

## Discussion

To our knowledge, this is the first review to synthesise the literature on women’s concerns about disclosing a high-risk cervical HPV infection to a sexual partner. The qualitative literature identified a range of concerns about disclosing HPV to a sexual partner. These concerns were partly because of the stigma associated with having an STI and the ways in which women anticipated their partners might respond. Some HPV-positive women used strategies to manage disclosure of their HPV diagnosis to a sexual partner, for example, focusing on having an abnormal screening result rather than HPV per se. The qualitative literature also found that women questioned how, when and to whom they should disclose their result. While quantitative and qualitative articles were included in the review, only one quantitative article was identified which found that over half of HPV-positive participants felt that disclosing their HPV-positive result was ‘risky’.

The results of this review suggest that some women feel anxious, worried and fearful about disclosing HPV to a sexual partner and described it as something they wished to avoid. These feelings were partly related to the stigma of having an STI and concerns about how others would respond to the disclosure of an HPV diagnosis. These findings are consistent with previous research with individuals diagnosed with other STIs such as HSV and chlamydia, where disclosure has been described as something that is difficult, fear-inducing^15^ and a considerable source of worry^16^ with feelings of stigma, shame and concerns about negative reactions from a sexual partner also reported.^15 16 20 22 23^ Although HPV is very common,^41^ one study that explored knowledge of HPV across the UK, USA and Australia found that less than half of the participants knew that most sexually active individuals would acquire HPV at some point in their life.^42^ Increasing knowledge of HPV and how common it is may help to reduce stigma around having the infection and reduce anxiety about disclosure. This review focused on women’s views about disclosing HPV to a sexual partner, but interestingly findings from the only quantitative study included in the review suggested that women may be more concerned about disclosing than men (60% vs 50% felt ‘disclosing is risky’, p=0.051). Future research could explore whether partners consider disclosure to be important.

During disclosure some women deliberately avoided mentioning HPV, focusing instead on their abnormal cytology or other aspects of their screening results. Managing the psychological implications of disclosure may be more challenging for women undergoing primary HPV testing who are told they are HPV-positive with normal cytology, given that HPV will be the only abnormal result they receive. They could, however, choose to focus on the normal cytology result. Following the introduction of primary HPV testing, it may be necessary to have additional support available for women. Healthcare professionals, particularly those carrying out cervical screening, are ideally placed to give brief information during screening which could help to mitigate the psychological impact of an HPV-positive result.

There are several advantages of primary HPV testing over cytology including increased sensitivity for detecting high-grade cervical abnormalities;^8–10^ and unlike cytological screening, HPV testing is not subjective so screening error rates are likely to be reduced. Despite the advantages of HPV testing, an essential criterion for any screening programme is that the benefit gained by individuals should outweigh the harms,^43^ therefore it is important to understand and address any adverse psychological consequences of testing HPV-positive. Alongside concerns about disclosing an HPV infection to a sexual partner, other research has found that receiving an HPV-positive result can lead to elevated anxiety, distress and concern about sexual relationships.^44 45^ However, research conducted in the context of the English cervical screening programme, where HPV testing was used as a triage to cytological screening, suggests that psychological effects are likely to be short-lived.^46^

Some women had questions about disclosing the infection to sexual partners, including whether disclosing was necessary. Disclosure is important for some STIs so that previous partners can be screened and treated for the infection if necessary, and future transmission of the infection can be prevented. However, while HPV is classified as an STI, it differs from other infections in that it does not usually need any treatment or cause any long-term problems. In addition, because most people will be infected with HPV at some point in their life,^5^ it is often difficult to determine where an HPV infection came from. Another systematic review,^27^ which explored the psychosexual impact of testing positive for hrHPV, identified concerns about where an HPV infection had come from as a common theme in the qualitative literature. Contact tracing for HPV is not routinely recommended by the WHO^25^ and therefore the decision to disclose HPV to a sexual partner is a personal choice. Cervical screening information materials should provide information about disclosing HPV to sexual partners to ensure that women are informed and that questions about disclosure do not cause any undue concern.

### Strengths and limitations

A strength of this review is that it was systematic and followed PRISMA guidelines. In addition, a broad search strategy was used with no date restrictions. It is possible that because of the range of terms that can be used to describe disclosure, some eligible studies may not have been identified in our search; however, we conducted forward and backward citation searching for all included studies to reduce the likelihood of this. Data were extracted by one author, with a second reviewer independently extracting data for 30% of the studies. It is possible that if the second reviewer extracted data from all the studies the results of the review could have changed; however, we feel this is unlikely as the agreement rate between reviewers was very good.

Only one quantitative paper was identified that reported disclosure-related outcomes, compared with six exploring the broader psychosexual impact of HPV, as identified by our related review.^27^ While the qualitative synthesis allowed us to highlight the range of different factors that contribute to women’s concerns about disclosure, we were unable to provide information on the percentage of women reporting each theme, as most of the papers included in the review did not quantify these. Assessing the prevalence and predictors of these concerns using quantitative methods is important and should be a priority for future research.

## Conclusions

This review synthesises the literature on women’s concerns about disclosing a high-risk cervical HPV infection to a sexual partner. The studies included in the review provide rich information about the range of concerns women have, the reasons for these concerns, and the questions women have about disclosing HPV to sexual partners. Increasing knowledge of HPV and providing clear information in screening information letters and leaflets about disclosing HPV to sexual partners may help women understand their screening result and minimise any unnecessary concern surrounding disclosure.

**Additional Educational Resources**

Human papillomavirus: https://www.nhs.uk/conditions/human-papilloma-virus-hpv/

NHS cervical screening - helping you decide: https://assets.publishing.service.gov.uk/government/uploads/system/uploads/attachment_data/file/827426/Cervical_screening_helping_you_decide_HPV.pdf

Screening, colposcopy, and cervical cancer: https://www.jostrust.org.uk/professionals
