# Supplementary material for: Concerns about disclosing a high-risk cervical human papillomavirus (HPV) infection to a sexual partner: a systematic review and thematic synthesis
Source: BMJ Sex Reprod Health. 2020 Jan 8;47(1):17–26. doi: 10.1136/bmjsrh-2019-200503 (PMC7815639; doi:10.1136/bmjsrh-2019-200503)
Supplement: Supplementary data [file bmjsrh-2019-200503supp001.pdf]

Supplementary Information 1
Search Strategy

	**MEDLINE, EMBASE, PsycINFO**	**CINAHL Plus**	**Web of Science**
**HPV**	1) HPV.mp.<br>2) "Human Papilloma Virus".mp.<br>3) "Human Papillomavirus".mp.<br>4) exp Papillomavirus Infections/<br>5) "Cervical intraepithelial neoplasia".mp.<br>6) Cervical Intraepithelial Neoplasia/<br>7) "Genital Warts".mp.<br>8) Condylomata Acuminata/<br>9) "Cervical Dysplasia".mp.<br>10) Uterine Cervical Dysplasia/	1) HPV<br>2) “Human Papilloma Virus”<br>3) “Human Papillomavirus”<br>4) MH “Papillomavirus Infections”<br>5) “Cervical intraepithelial neoplasia”<br>6) MH “Cervical Intraepithelial Neoplasia”<br>7) “Genital Warts”<br>8) MH “Warts, Veneral”<br>9) “Cervical Dysplasia”	1) HPV<br>2) “Human Papilloma Virus”<br>3) “Human Papillomavirus”<br>4) “Cervical Intraepithelial Neoplasia”<br>5) “Genital Warts”<br>6) “Cervical Dysplasia”
**PSYCHOSEXUAL OUTCOMES**	12) Psychosexual.mp.<br>13) Psychosocial.mp.<br>14) Psych*.mp.<br>15) “Quality of Life”.mp<br>16) "Quality of Life"/<br>17) Sexual Dysfunctions, Psychological/<br>18) “Sex* Impact”<br>19) Disclos*.mp.<br>20) Disclosure/	11) Psychosexual<br>12) Psychosocial<br>13) Psych*<br>14) “Quality of Life”<br>15) MH “Quality of Life”<br>16) (MH “Sexual Dysfunction, Female”) OR (MH “Psychosexual Disorders”)<br>17) “Sex* Impact”<br>18) Disclos*	8) Psychosexual<br>9) Psychosocial<br>10) Psych*<br>11) “Quality of Life”<br>12) “Sex* Impact”<br>13) “Sex* Function*”<br>14) Disclos*
**SEARCH COMBINATIONS**	11) 1 or 2 or 3 or 4 or 5 or 6 or 7 or 8 or 9 or 10<br><br>21) 12 or 13 or 14 or 15 or 16 or 17 or 18 or 19 or 20	10) 1 or 2 or 3 or 4 or 5 or 6 or 7 or 8 or 9<br><br>19) 11 or 12 or 13 or 14 or 15 or 16 or 17 or 18	7) 1 or 2 or 3 or 4 or 5 or 6<br><br>14) 8 or 9 or 10 or 11 or 12 or 13
**INCLUDED**	11 and 21	10 and 19	7 and 14
