# Supplementary material for: Concerns about disclosing a high-risk cervical human papillomavirus (HPV) infection to a sexual partner: a systematic review and thematic synthesis
Source: BMJ Sex Reprod Health. 2020 Jan 8;47(1):17–26. doi: 10.1136/bmjsrh-2019-200503 (PMC7815639; doi:10.1136/bmjsrh-2019-200503)
Supplement: Supplementary data [file bmjsrh-2019-200503supp003.pdf]

Supplementary Information 2

Data Extraction Form

Concerns about disclosing a high-risk cervical human papillomavirus (HPV) infection to a sexual partner: a systematic review and thematic synthesis.

ID Number (on Excel spreadsheet)	
Date form completed	
Authors	
Title	
Journal	
Year	
Volume	
Issue	
Pages	
**Participants**	
HPV status determined?	YES NO
Type of HPV (HR, HR and LR, unsure)	
Number of participants	
Age range of participants	
Gender of participants	
Other relevant sociodemographics	
**Methods**	
Study design	
Aim of study	
Recruitment method	
Recruitment setting	
Outcomes measured	
Method(s) of analysis	
**Results**	
(Psycho)sexual outcomes reported?	YES NO
If yes, give summary of results	
Disclosure outcomes reported?	YES NO
If yes, give summary of results	
Other notes	
