# Supplementary material for: Concerns about disclosing a high-risk cervical human papillomavirus (HPV) infection to a sexual partner: a systematic review and thematic synthesis
Source: BMJ Sex Reprod Health. 2020 Jan 8;47(1):17–26. doi: 10.1136/bmjsrh-2019-200503 (PMC7815639; doi:10.1136/bmjsrh-2019-200503)
Supplement: Supplementary data [file bmjsrh-2019-200503supp004.pdf]

Supplementary Information 4
Quality Appraisal Checklist – Qualitative Studies

Concerns about disclosing a high-risk cervical human papillomavirus (HPV) infection to a sexual partner: a systematic review and thematic synthesis.

**ID Number (on Excel spreadsheet)**		
**Date form completed**		
**Assessed by**		
**Authors**		
**Title**		
**Journal**		
**Year**		
**Volume**		
**Issue**		
**Pages**		
**THEORETICAL APPROACH**		
**Is a qualitative approach appropriate?**<br>*For example:*<br>• *Does the research question seek to understand processes or structures, or illuminate subjective experiences or meanings?*<br>• *Could a quantitative approach better have addressed the research question?*	Appropriate<br>Inappropriate<br>Not sure	Comments:
**Is the study clear in what it seeks to do?**<br>*For example:*<br>• *Is the purpose of the study discussed – aims/objectives/research question/s?*<br>• *Is there adequate/appropriate reference to the literature?*<br>• *Are underpinning values/assumptions/theory discussed?*	Clear<br>Unclear<br>Mixed	Comments:
**STUDY DESIGN**		
**How defensible/rigorous is the research design/methodology?**<br>*For example:*<br>• *Is the design appropriate to the research question?*<br>• *Is a rationale given for using a qualitative approach?*<br>• *Are there clear accounts of the rationale/justification for the sampling, data collection and*	Defensible<br>Indefensible<br>Not sure	Comments:

data analysis techniques used?<br>• *Is the selection of cases/sampling strategy theoretically justified?*		
**DATA COLLECTION**		
**How well was the data collection carried out?**<br>*For example:*<br>• *Are the data collection methods clearly described?*<br>• *Were the appropriate data collected to address the research question?*<br>*Was the data collection and record keeping systematic?*	Appropriately<br>Inappropriately<br>Not sure/inadequately reported	Comments:
**Is the context clearly described?**<br>*For example:*<br>• *Are the characteristics of the participants and settings clearly defined?*<br>• *Were observations made in a sufficient variety of circumstances*<br>• *Was context bias considered*	Clear<br>Unclear<br>Not sure	Comments:
**Were the methods reliable?**<br>*For example:*<br>• *Was data collected by more than 1 method?*<br>• *Is there justification for triangulation, or for not triangulating?*<br>• *Do the methods investigate what they claim to?*	Reliable<br>Unreliable<br>Not sure	Comments:
**ANALYSIS**		
**Is the data analysis sufficiently rigorous?**<br>*For example:*<br>• *Is the procedure explicit – i.e. is it clear how the data was analysed to arrive at the results?*<br>• *How systematic is the analysis, is the procedure reliable/dependable?*<br>• *Is it clear how the themes and concepts were derived from the data?*	Rigorous<br>Not rigorous<br>Not sure/not reported	Comments:
**Is the data 'rich'?**<br>*For example:*<br>• *How well are the contexts of the data described?*	Rich<br>Poor<br>Not sure/not reported	Comments:

• *Has the diversity of perspective and content been explored?*<br>• *How well has the detail and depth been demonstrated?*<br>• *Are responses compared and contrasted across groups/sites?*		
**Is the analysis reliable?**<br>*For example:*<br>• *Did more than 1 researcher theme and code transcripts/data?*<br>• *If so, how were differences resolved?*<br>• *Did participants feedback on the transcripts/data if possible and relevant?*<br>• *Were negative/discrepant results addressed or ignored?*	Reliable<br>Unreliable<br>Not sure/not reported	Comments:
**Are the findings convincing?**<br>*For example:*<br>• *Are the findings clearly presented?*<br>• *Are the findings internally coherent?*<br>• *Are extracts from the original data included?*<br>• *Are the data appropriately referenced?*<br>• *Is the reporting clear and coherent?*	Convincing<br>Not convincing<br>Not sure	Comments:
**Are the findings relevant to the aims of the study?**	Relevant<br>Irrelevant<br>Partially relevant	Comments:
**Conclusions**<br>*For example:*<br>• *How clear are the links between data, interpretation and conclusions?*<br>• *Are the conclusions plausible and coherent?*<br>• *Have alternative explanations been explored and discounted?*<br>• *Does this enhance understanding of the research topic?*<br>• *Are the implications of the research clearly defined?*<br>• *Is there adequate discussion of any limitations encountered?*	Adequate<br>Inadequate<br>Not sure	Comments:
**Ethics**		

**How clear and coherent is the reporting of ethics?**<br>*For example:*<br>• *Have ethical issues been taken into consideration?*<br>• *Are they adequately discussed e.g. do they address consent and anonymity?*<br>• *Have the consequences of the research been considered i.e. raising expectations, changing behaviour?*<br>• *Was the study approved by an ethics committee?*	Appropriate<br>Inappropriate<br>Not sure/not reported	Comments:
**Overall assessment**		
**As far as can be ascertained from the paper, how well was the study conducted? (see guidance notes)**	++<br>+<br>−	Comments:

++ All or most of the checklist criteria have been fulfilled, where they have not been fulfilled the conclusions are very unlikely to alter.

+ Some of the checklist criteria have been fulfilled, where they have not been fulfilled, or not adequately described, the conclusions are unlikely to alter.

– Few or no checklist criteria have been fulfilled and the conclusions are likely or very likely to alter.
